# Adequate Patient’s Outcome Achieved with Short Immunoglobulin Replacement Intervals in Severe Antibody Deficiencies

**DOI:** 10.1007/s10875-014-0081-9

**Published:** 2014-07-22

**Authors:** Cinzia Milito, Federica Pulvirenti, Anna Maria Pesce, Maria Anna Digiulio, Franco Pandolfi, Marcella Visentini, Isabella Quinti

**Affiliations:** 1 Department of Molecular Medicine, Sapienza University of Rome, Viale dell’Università 37, Rome, Italy; 2 Department of Internal Medicine, Catholic University, Rome, Italy

**Keywords:** Primary antibody deficiencies, patients’ outcomes, replacement therapy, immunoglobulins, intervals, dosages, cost

## Abstract

**Purpose:**

The optimal immune globulin replacement dosages required over time to minimize infection risks in patients with Primary Antibody Deficiencies are not definitely established. As with many interventions, there may be specific subgroups of patients who are more likely to benefit from treatment with higher or lower dosages. The aim of the study was to verify the efficacy of a rationale for individualized immune globulin utilization and to elucidate the effects of care on patient outcome.

**Methods:**

Single centre interventional study on 108 patients with Primary Antibody Deficiencies. The objective was to determine for each patient the best interval between immune globulins administration in order to: • Keep IgG trough levels >500 mg/dL, • Minimize of major infections (pneumonias and infections requiring hospitalization), • Minimize of adverse events (AE).

**Results:**

Ninthly eight per cent of patients achieved the objective of the study. Patients who had low switched memory B cells and low IgA serum levels and/or are affected by bronchiectasis and/or enteropathy and/or continued to experience adverse events despite pre-medications, achieved the study objective by shortening the administration intervals to 2-weeks or to 1-week without the need to increase the monthly cumulative immunoglobulin dosage and its relative cost. The adverse events were reduced by administrating low Ig dosages in a single setting. Patients without risk factors achieved the study objective with immune globulin replacement administered with the widely used interval of 3 or 4 weeks.

**Conclusions:**

The exact timing and optimal immunoglobulin prophylaxis regimen might be tailored according to clinical and immunological markers.

## Introduction

Immune globulin (Ig) replacement is the standard therapy for primary antibody deficiencies (PAD) aiming to replace the missing antibodies and thereby to prevent recurrent infections [1–3]. The optimal Ig dosages required over time to minimize infection risk are not definitely established with a consequent wide variation in treatment practices [4–6]. Debate continues regarding the exact timing and the optimal prophylaxis regimen [7–9], knowing that the system of care is itself an important determinant of patient outcomes. Unfortunately, many health care delivery systems are subjected to economic pressures. Individualized medicine and personalized health research presents methodological challenges [10]. In PAD different options have been explored to establish how we should individualize Ig replacement therapy [11–13]. As with many interventions, there may be specific subgroups of patients [14] who are more likely to benefit from treatment with higher or lower dosages of Ig.

In PAD, we have previously identified [15] a clinical phenotype characterized by a high risk of acquiring infections: patients who had low IgG and IgA levels at diagnosis; patients who had IgA level <7 mg/dL and who had bronchiectasis [16]. These data confirmed previous observations showing that the loss of function of memory B cells seems to represent the major cause of PAD-associated clinical conditions [17–20]. Here we show the results of an interventional single centre study on 108 PAD patients aimed to verify the efficacy of a new rationale for an individualized intravenous Ig (IVIG) utilization in the treatment of symptomatic PADs, Common Variable Immune Deficiencies (CVIDs) and X-linked Agammaglobulinemia (XLA). Our hypothesis was that an adequate patient’s outcome could be achieved by shorting the administration intervals to 1- or 2-weeks without the need to greatly increase the monthly cumulative Ig dosage in those PAD patients: 1) who present an infectious risk profile; 2) who are affected by bronchiectasis and/or enteropathy; 3) who continue to have adverse events (AE) despite pre-medication.

## Materials and Methods

### Study Design

**Fig. 1:**
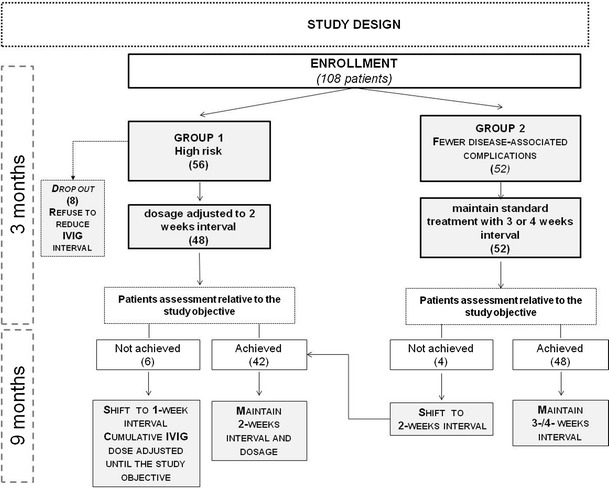
Flow chart of the study design. Numbers of patients enrolled in the different arms of the study are shown

prospective non-randomized interventional single centre study in PAD patients over 1 year period. Study design was shown in Fig. 1.

### Aim of the Study

to determine for each patient the optimal infusion frequency of immune globulins and the Ig cumulative monthly dosage in order to achieve the following goals:

- maintenance of IgG trough levels >500 mg/dL;
- minimization of major infections (pneumonias and infections requiring hospitalization);
- minimization of AE IVIG-related.

### Group Definition

Patients on IVIG were enrolled into two groups according to the following parameters: serum IgG trough levels; serum IgA levels; presence of bronchiectasis; presence of enteropathy; positive history for AE IVIG-related.

- *Group 1 (high risk):* patients receiving IVIG replacement every 3 or 4 weeks who had one of the following:
  - AEs despite premedication by steroids (hydrocortisone 100–250 mg), paracetamol and anti-hystamine drugs;
  - IgG trough levels <500 mg/dL
  - IgA <7 mg/dL
  - Bronchiectasis
  - Enteropathy.
- *Group 2 (fewer disease-associated complications)*: patients receiving IVIG replacement every 3–4 weeks who did not meet the criteria listed above.

Patients of group 1 changed to fortnightly infusion with a constant monthly dosage; patients of group 2 continued to follow the usual pattern of Ig administration.

After 3 months, an assessment relative to the study objective was performed: patients of group 1 who did not achieve the objective changed from fortnightly to weekly infusions; the cumulative monthly dosage was increased in order to reach the objective (1-week); patients of group 1 who achieved the objective remained at the established 2 weeks interval (2-weeks interval) and did not increase the cumulative monthly Ig dose. Patients of group 2 who did not achieved the objective of the study were changed to fortnightly infusion; patients of group 2 who achieved the objective remained at the established 3 or 4 weeks intervals.

Data of 13 PAD patients receiving subcutaneous (SCIG) replacement at the study time were also collected. These patients continued their schedule of SCIG administration during the study time.

### Patients

We enrolled all 108 patients with primary antibody deficiency (PAD) attending our Reference Centre for Primary Immune Deficiencies. Patients were diagnosed according to the ESID/PAGID criteria for XLA and CVIDs [21]. Ten patients had a definitive diagnosis of XLA based on <2 % of peripheral B cells and mutations of the Btk gene; 98 patients had a diagnosis of CVIDs based on IgG <500 mg/dL, IgA 2 standard deviation (SD) below age-specific reference range, age onset >4 years, poor response to vaccines and exclusion of other causes of hypogammaglobulinemia.

A detailed sets of data was available since all patients with a diagnosis of CVID and XLA have been regularly followed up in our centre according to the Italian guide-lines (www.aieop.org) and their clinical and immunological data have been regularly collected in a national database once a year. The data set included: age, date of PAD diagnosis, serum IgG, IgA and IgM levels determined every 3 months, clinical manifestations, route, doses and intervals of Ig replacement, occurrence of AE. At the time of this interventional study, we continued to follow our guidelines collecting for each patient: full blood counts, chemistry and serum Ig every 3 months; clinical manifestations once a month. Lymphocyte subsets phenotype analysis were repeated once at time of the study. Route, dosage and interval of Ig replacement was recorded once a month. High-resolution chest Computerised Tomography (HRCT) scans was performed once every four years in all patients according to National guidelines (www.aieop.org).

All patients have been IVIG or SCIG replacement for at least 5 years. All participants provided written informed consent. The study protocol was approved by the Institutional Review Board at University Sapienza of Rome.

### Statistical Analysis

Parametric and non-parametric data was presented as mean ± SD or range, as indicated. For comparison between groups, the Student *T* test and Mann–Whitney test were used for parametric or non-parametric datasets. We used SAS, JMP8 version (SAS Institute, Cary, North Carolina), for all statistical analyses. Results were expressed as hazard ratios (HR) with 95 % CIs and p values. A p value of <0.05 was taken as the threshold of statistical significance.

## Results

### Intervals Between Administrations and Dosages of the Ig Replacement

Based on the hypothesis that an IgG trough level might be maintained at higher levels by decreasing the infusion frequency without the need to greatly increase the cumulative monthly Ig dosage, we established for each patient the optimal Ig replacement interval necessary to keep the patient free of major infections and free of AE. Clinical and immunological data of patients grouped according to the IVIG administration intervals were shown in Table 1. Data of patients on SCIG replacement were also shown.

**Table 1:** Clinical and Immunological data of 108 PAD patients grouped according to the IVIG administration intervals. Data on 13 patients treated with SCIG are also shown

Intervals	Number of patients	Monthly Ig dose (mg/kg/month)	IgG trough levels (mg/dL)	CVID/XLA	IgA levels (mg/dL)	B cells (cells/mm3)	Switched memory B cells (%)	CD4+ T cells (cells/mm3)	Bronchiectasis	Enteropathy	IgA <7 (mg/dL)	AE
1-week	6	578 ± 70	595 ± 88	4/2	1 ± 2.5	63 ± 60	0.7 ± 1	518 ± 276	6	5	6	5
2-weeks	46	361 ± 103	693 ± 131	37/8	4 ± 4	133 ± 117	2.6 ± 3.7	619 ± 407	33	14	39	12
3-weeks	31	230 ± 71	657 ± 103	31/0	9 ± 1	177 ± 140	3.5 ± 3.0	567 ± 213	0	0	0	0
4-weeks	17	210 ± 93	615 ± 84	17/0	9.5 ± 2	105 ± 89	8 ± 4.6	541 ± 179	0	0	0	0
SCIG	13	323 ± 91	641 ± 164	16/0	20 ± 19	341 ± 222	7.5 ± 4.9	486 ± 262	2	0	2	1

### Patients Under IVIG Administered at Short Interval Times: 1 Week or 2 Weeks Intervals

The interval between IVIG administrations was established every 2 weeks in 56/60 patients who fulfilled the inclusion criteria of group 1 (“*high risk*”). Eight patients refused to shorten the interval between IVIG administrations for logistical reasons and/or for poor compliance, so they were not included in the analysis. Thus, the total number of patients included this group was 48 patients.

After 3 months from the enrolment, a patient assessment relative to the study objective was perform: 42/48 patients achieved the objective and continued to receive IVIG infusion fortnightly (2-weeks). 6/48 patients did not achieve the objective: 2 patients couldn’t maintain IgG trough levels >500 mg/dL because of severe enteropathy and 4 patients experienced AEs post-IVIG (back pain, chills and fever) despite administrations of steroids, paracetamol and anti-histamine drugs before IVIG infusion; these patients could not tolerate more than 5–7.5 g of immune globulins per setting. For this reason, all six reduced the IVIG administration interval to every week (1-week). Immunological and clinical data of patients receiving IVIG at 1-week were show in Table 2.

**Table 2:** Clinical and immunological data of the six patients who received IVIG replacement weekly

patient	Monthly Ig dosage	IgG TL (mg/dL)	Bronchiectasis	Severe Entheropathy	IgA < 7 mg/dl	AE
1	500	590	yes	no	yes	yes
2	570	750	yes	no	yes	yes
3	690	540	yes	no	yes	yes
4	510	640	yes	no	yes	yes
5	660*	510	yes	yes	yes	no
6	600*	540	yes	yes	yes	no

*: Cumulative monthly dosage administered by IVIG (200 mg/kg/month) and SCIG (100 mg/kg/week)

Four additional patients from group 2 who did not achieve the study objective after 3 months changed to fortnightly infusion.

**Fig. 2:**
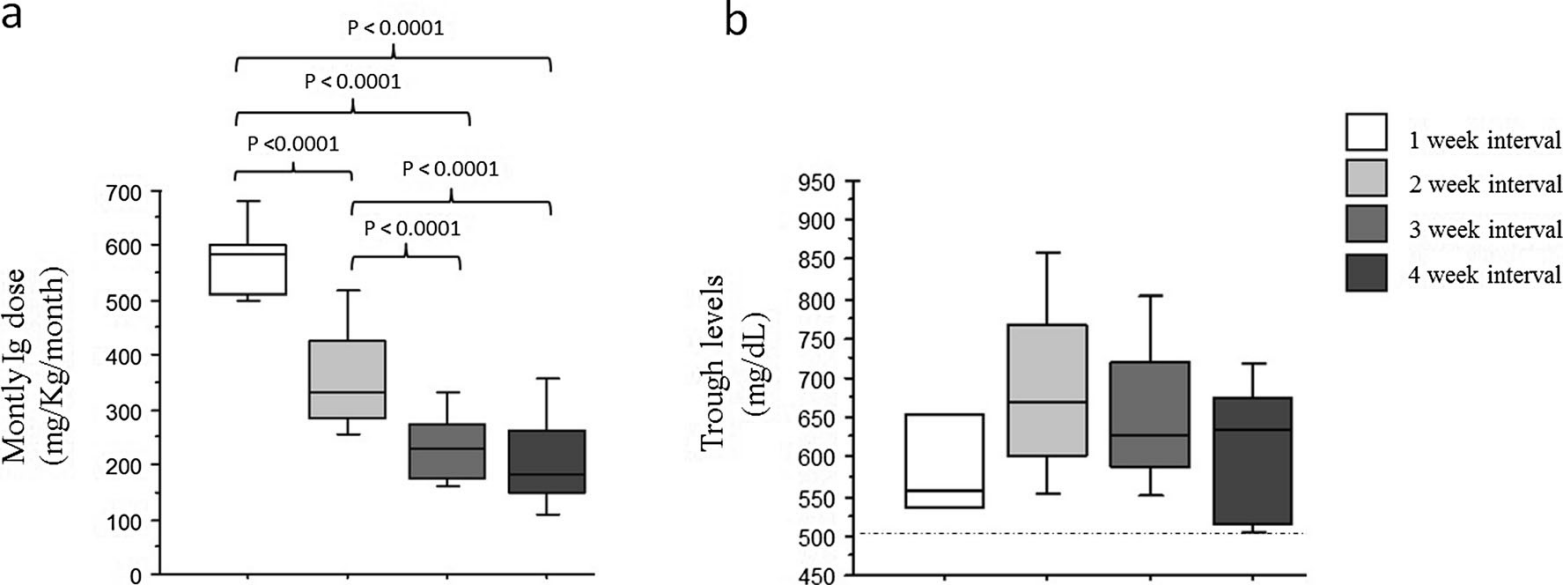
IgG trough levels and cumulative immune globulin monthly dosages. Cumulative monthly Ig dose (mg/kg/month) administered (a) and serum IgG trough levels (mg/dL) (b) in PAD patients grouped according to the replacement intervals of IVIG administration: 1-week, 2-weeks, 3-weeks, 4-weeks. Statistical differences between groups are shown

In summary, at the end of the study six patients received IVIG weekly (*1-week*) and 46 patients every two weeks (*2-weeks*). The cumulative mean monthly Ig dosage differed among the groups: 578 ± 70 mg/kg/month (1-week) and 361 ± 103 mg/kg/month (2-weeks) (p <0.0001, Fig. 2.a). Two patients affected by a severe enteropathy maintained IgG trough levels >500 mg/dL by only administering both IVIG (one infusion/month at 200 mg/kg/month) and SCIG (100 mg/kg/week). Patients at 1-week interval had more frequently enteropathy (5/6 *vs* 14/46 p = 0.01) and AEs post-IVIG (5/6 *vs* 12/46 p = 0.005) in comparison to patients at *2-weeks* interval. No differences were observed on the number of patients with bronchiectasis or IgA levels <7 mg/dL between these groups.

### Patients Under IVIG Administered at Usual Interval Times: 3 Week or 4 Weeks Intervals

Fifty-two out of 108 patients received IVIG administrations every 3 weeks or 4 weeks; all these patients had a diagnosis of CVID. None these patients had the clinical phenotype yet identified as a severe PAD phenotype, that is characterized by low IgG and IgA levels at diagnosis, IgA level <7 mg/dl, <2 % of switched memory B cells, presence of bronchiectasis, and/or enteropathy [15, 16, 22, 23]. Thirty-five patients received IVIG every 3 weeks and 17 patients received IVIG every 4 weeks.

As explained above after 3 months, 4 patients were changed to fortnightly infusion. In summary, at the end of the study, 31 patients received IVIG every 3 week (*3-week*) and 17 patients every four weeks (*4-weeks*); all these patients achieved the study objective. The cumulative mean monthly Ig dosage did not differ between the 3-weeks and the 4-weeks intervals: 230 ± 71 mg/kg/month (3-weeks) and 210 ± 93 mg/kg/month (4-weeks) (Fig. 2a). The cumulative mean monthly Ig dosages in these patients were lower than the cumulative mean monthly Ig dosages administered in patients receiving IVIG weekly or fortnightly (*p values* shown in Fig.2a) and lower than the cumulative monthly dosage administered in patients on SCIG (323 ± 91 mg/kg/month).

### Immunological Phenotype of Patients Treated with Longer or Shorter Intervals

**Fig. 3:**
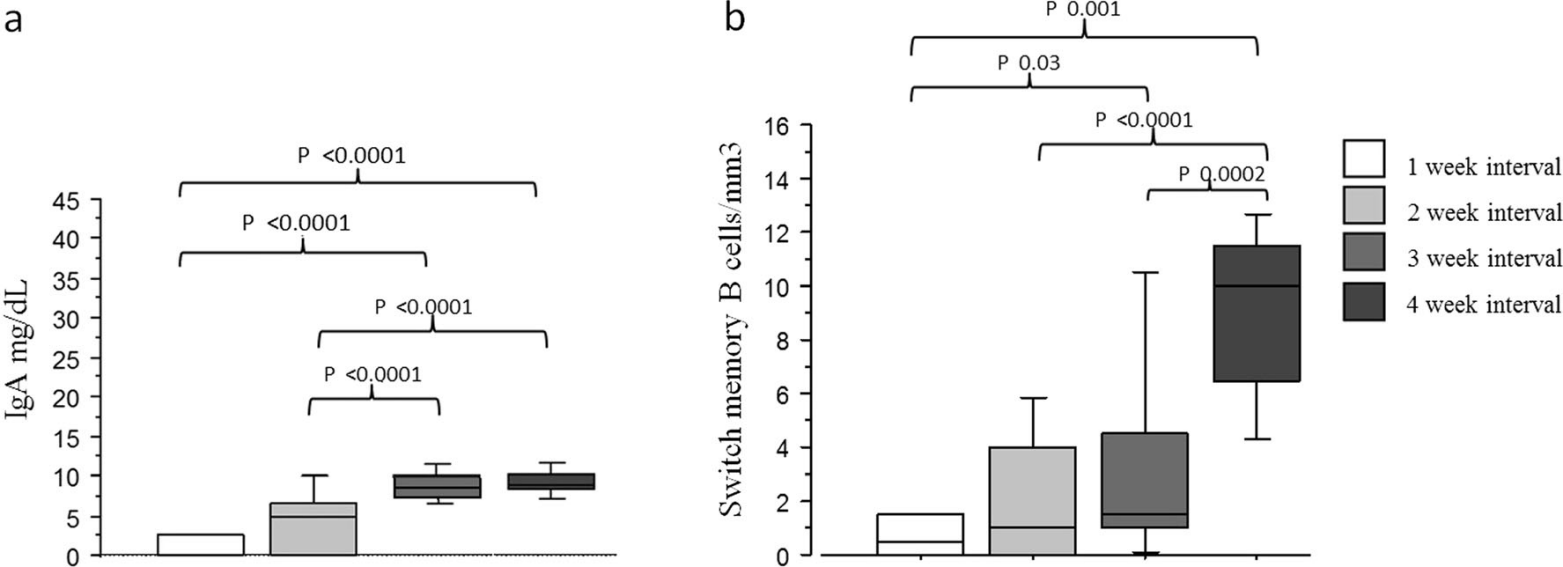
Immunological phenotype of patients treated at different Ig administration intervals. (a) IgA serum levels; (b) percentages of peripheral blood switched B memory B cells (CD19 + CD27 + IgM-IgD-) in PAD patients grouped according to the replacement intervals of IVIG administration: 1-week, 2-weeks, 3-weeks, 4-weeks. Statistical differences between groups are shown

According to the study design, serum IgG trough levels in the four groups were greater than 500 mg/dL (Fig. 2b and Table 1): 595 ± 88 mg/dL (1-week); 693 ± 131 mg/dL (2-weeks); 657 ± 103 mg/dL (3-weeks); 615 ± 84 mg/dL (4-weeks); in the SCIG group the IgG levels were 641 ± 164 mg/dL. Mean IgA serum levels reflected the inclusion criteria. IgA levels differed between the groups: 1 ± 2.5 mg/dL (1-week); 4 ± 4 mg/dL (2-weeks); 9 ± 1 mg/dL (3-weeks); 9.5 ± 2 mg/dL (4-weeks) (*p values* are shown in Fig.3a).

Serum IgM and serum IgG at diagnosis did not differed between the groups.

No significant differences were found in the peripheral B lymphocyte frequencies within the four groups. Percentages of peripheral blood switched B memory cells differed between the groups: 1-week: 0.75 ± 1 %; 2-weeks: 2.6 ± 3.7 %; 3-weeks: 3.5 ± 3 %; 4-weeks 8 ± 4.6 % (*p values* were shown in Fig. 3.b). Notably, in our cohort, the prevalence of CVIDs patients with a severe defect of switched memory B cells in the peripheral blood reflects that described in the European cohort in Euroclass paper [24].

No significant differences in the frequencies of peripheral blood CD3 + CD4+ cells were observed within the four groups, despite a lower frequency observed in the group of patients who received IVIG at 1-week interval: 518 ± 276/mm3 (1-week); 619 ± 407/mm3 (2-weeks); 567 ± 213/mm3 (3-weeks); 541 ± 179/mm3 (4-weeks). B cell subsets showed a high variability in the group of patients on SCIG (Table 1). This reflected the different criteria we adopted for the SCIG choice, mainly linked to logistical reasons.

## Discussion

The demonstrated success of Ig prophylaxis depends predominantly on maintaining an adequate protection against infections. Most papers on Ig replacement have concluded that the therapy should be individualized [4–9, 11–13, 25]. Our study points to the need to elucidate the effects of care on patient outcomes, in order to identify what works in which setting and under what conditions. According to international guidelines the Ig monthly dose of 300–600 mg/kg body weight should be administer intravenously every 3 or 4 weeks and subcutaneously once/twice a week. The trend over the past years has been to increase the monthly cumulative doses [8, 9, 11, 25]. These general rules might not be optimal for all PAD patients, due to high clinical and immunological heterogeneity of the underlying diseases [14]. Moreover, the cost inherent to the need to increase in *all* PAD patients the trough IgG level might not be justified and sustainable for many countries. This strategy may require an increase on the monthly Ig dose with the consequent increase of the Ig cost. The pharmacokinetics of IgG and specific antibodies demonstrated a different half-life in patients treated at different intervals between infusions [26]. Trough levels of antibodies with the lowest specific antibody concentrations rise if regular infusions are given and the actual trough levels in a regularly infused patient are likely to be greater than the levels of specific IgG measured by ELISA in the IVIG preparations [27].

Thus, an important determinant of the efficacy of Ig prophylaxis is the length of time an individual spends with a lower IgG level as it was show in PAD patients treated with SCIG [28]. This time is more dependent on the patient’s IgG half-life and the frequency of dosing than on the amount of the dose infused. In the clinical practice, it would be ideal to perform a pharmacokinetics (PK) study in all patients. However, this would require a significant commitment in time from the patient. This practical drawback has limited the use of PK information in clinical practice. Different alternative options have been attempt. The Oxford choice, for example, was to increase the IVIG dose by 0.15 g/kg/month when patients present with a serious infection, or 3 or more moderate infections over a year [13]. This recommendation could be an alternative for patients who have persistent infections; although other factors such as protein loosing conditions, airway and intestinal inflammation, need to be assessed when defining the individual Ig schedule of treatment. Moreover, in PAD, several lines of experimental evidence gathered recently [29] provided a basis for an active role for IVIG in immunomodulation beside the main role to replace the missing antibodies. IVIG has an active role in regulating autoimmune and inflammatory responses through modulating B and other cells functions [30, 31]. These new findings might help to explain the different results found in trails aimed to establish the clinical outcome of Ig replacement in PAD patients. It is possible that some of the positive effects observed in patients treated with higher doses are not dependent only on the prophylactic role of immune globulins but also on the anti-inflammatory ones [32]. However, higher Ig dosages can potentially cause side effects [33]. The results of this study showed that in patients with fewer CVIDs-associated complications the Ig replacement could be administer with the widely used interval of 3 or 4 weeks, even administering low Ig replacement dosages. On the opposite, in patients with a severe clinical and immunological phenotype the protective effect might be achieved by lowering the interval between administrations to 2 weeks and in few cases to 1 week, without increasing the cumulative monthly Ig dosage and its relative cost. We also demonstrated that the occurrence of adverse events in patients who do not tolerate Ig despite pre-medication might be greatly reduce by decreasing the Ig doses administered in one setting with a reduced interval between administrations.

This workup may help to expand access to prophylaxis in healthcare systems with limited resources and potentially improve patient outcomes. The extra day/month used for Ig therapy might be consider costly both for patients and for health care. However, in Italy, patients with severe chronic diseases benefit of 3 days off work/month for treatments without any loss of their salary.

Health care delivery systems are quickly changing in response to economic pressures and concerns about quality of care. The system of care is itself an important determinant of patient outcomes. The promise of individualized medicine has launched a huge research enterprise to explore the personal characteristics that influence responses to therapy [10]. We are challenging our beliefs through real data coming from our current practice using a latent therapeutic demand for Ig.

Our results are in agreement with the subcutaneous Ig replacement data. However, differently than the subcutaneous administration, the intravenous administration might allow to maintain the protective and immune-modulatory effects due to the serum IgG peak reached at the time of each administration. In patients who did not achieve a satisfactory efficacy, both intravenous and subcutaneous Ig might be administered. We concluded that the suggested protective high trough IgG level should not be considered a general goal and only large prospective multi-centre studies might help to clarify which CVIDs subgroups are at high infection risk. More work is necessary to define which PAD-associated conditions need a higher or lower monthly Ig dosage and the best interval between administrations. However, the considerations expressed above have a vast potential to ameliorate the clinical practice of Ig replacement treatment.
